# Profiling Speech and Pausing in Amyotrophic Lateral Sclerosis (ALS) and Frontotemporal Dementia (FTD)

**DOI:** 10.1371/journal.pone.0147573

**Published:** 2016-01-20

**Authors:** Yana Yunusova, Naida L. Graham, Sanjana Shellikeri, Kent Phuong, Madhura Kulkarni, Elizabeth Rochon, David F. Tang-Wai, Tiffany W. Chow, Sandra E. Black, Lorne H. Zinman, Jordan R. Green

**Affiliations:** 1 Department of Speech-Language Pathology, University of Toronto, Toronto, Ontario, Canada; 2 Sunnybrook Research Institute, Toronto, Ontario, Canada; 3 University Health Network—Toronto Rehabilitation Institute, Toronto, Ontario, Canada; 4 Department of Medicine (Neurology), University of Toronto, Toronto, Ontario, Canada; 5 Division of Neurology, Toronto Western Hospital, University Health Network, Toronto, Ontario, Canada; 6 Rotman Research Institute, Toronto, Ontario, Canada; 7 L.C. Campbell Cognitive Neurology Research Unit, Sunnybrook Health Sciences Centre, Toronto, Ontario, Canada; 8 Sunnybrook Health Sciences Centre, University of Toronto, Toronto, Ontario, Canada; 9 MGH Institute of Health Professions, Boston, Massachusetts, United States of America; University of Ulm, GERMANY

## Abstract

**Objective:**

This study examines reading aloud in patients with amyotrophic lateral sclerosis (ALS) and those with frontotemporal dementia (FTD) in order to determine whether differences in patterns of speaking and pausing exist between patients with primary motor vs. primary cognitive-linguistic deficits, and in contrast to healthy controls.

**Design:**

136 participants were included in the study: 33 controls, 85 patients with ALS, and 18 patients with either the behavioural variant of FTD (FTD-BV) or progressive nonfluent aphasia (FTD-PNFA). Participants with ALS were further divided into 4 non-overlapping subgroups—mild, respiratory, bulbar (with oral-motor deficit) and bulbar-respiratory—based on the presence and severity of motor bulbar or respiratory signs. All participants read a passage aloud. Custom-made software was used to perform speech and pause analyses, and this provided measures of speaking and articulatory rates, duration of speech, and number and duration of pauses. These measures were statistically compared in different subgroups of patients.

**Results:**

The results revealed clear differences between patient groups and healthy controls on the passage reading task. A speech-based motor function measure (i.e., articulatory rate) was able to distinguish patients with bulbar ALS or FTD-PNFA from those with respiratory ALS or FTD-BV. Distinguishing the disordered groups proved challenging based on the pausing measures.

**Conclusions and Relevance:**

This study demonstrated the use of speech measures in the identification of those with an oral-motor deficit, and showed the usefulness of performing a relatively simple reading test to assess speech versus pause behaviors across the ALS—FTD disease continuum. The findings also suggest that motor speech assessment should be performed as part of the diagnostic workup for patients with FTD.

## Introduction

Pausing while speaking provides insight into the multiple stages of spoken word production. Adults, who are fluent speakers, and readers, speak rather rapidly and pause in speech following a predictable pattern. Physiologically, they pause to take a breath. However, they are also able to precisely control the intake of breath in order to make conceptual, syntactic, and lexical decisions, as well as to convey psychological states and emotional information [1–5]. In addition to the cognitive load and the complexity of the linguistic message, speech breathing and pausing are affected by motor processes that are associated with the planning and execution of utterances (e.g., speaking rate and utterance length variation) [6–9]. Consequentially, conditions associated with changes in motor control and/or cognitive-linguistic deficits may result in predictable changes in speaking and/or pausing, and the tracking of these resultant changes could potentially serve a role in the diagnosis of such conditions.

This study examines pausing while reading aloud in patients with amyotrophic lateral sclerosis (ALS) and those with frontotemporal dementia (FTD), in order to determine whether differences in patterns of speaking and pausing exist between patients with primary motor vs. primary cognitive-linguistic deficits, and in contrast to healthy controls. In ALS, the motor neurons are affected in the brain, brainstem and spinal cord, and there is a progressive loss of muscle control and strength. Oral motor function (e.g., contraction of musculature during speaking and swallowing) is impaired in the *bulbar* form of the disease. Although motor symptoms predominate, 10% of patients with ALS exhibit symptoms of FTD, and up to 50% show signs of impairment across various cognitive and language domains, which may not be clinically apparent, but which can be identified using detailed neuropsychological testing [10–12]. In FTD, the disease affects the frontal and/or temporal lobes of the brain, with four main variants identified. In the behavioural variant (FTD-BV), there are progressive changes in behaviour and frontal/executive function, while language abilities are usually preserved [13]. The remaining types of FTD are all variants of Primary Progressive Aphasia (PPA) and are characterized by a primary language impairment [14]. Of a particular interest is the nonfluent variant, also known as progressive nonfluent aphasia (FTD-PNFA), which presents with slow and labored speech, grammatical errors and apraxia of speech (AOS)—a motor speech disorder—characterized by slow speaking rate, prosodic abnormalities, and articulatory problems. ALS, FTD-PNFA and FTD-BV represent a continuum with respect to the extent of motor versus cognitive involvement. Whereas ALS diagnosis is based on clear motor involvement, FTD-PNFA patients can show both cognitive-linguistic and motor speech features, while patients with FTD-BV must show a clearly defined cognitive (frontal/executive) deficit in order to meet diagnostic criteria, and do not usually exhibit a motor impairment. Because these neurodegenerative conditions are thought to present along a clinical, pathological and genetic continuum [10], [15], identification of primary motor versus primary cognitive-linguistic effects on common tasks that involve speaking or reading aloud can be important, not only for diagnosis, but also for disease monitoring.

### Speaking and pausing in ALS

The bulbar form of ALS commonly affects laryngeal, velopharyngeal, and oral articulatory musculature, resulting in significant deficits in phonatory, resonatory and articulatory functions [16]. Across these motor functions, the presence of bulbar impairments is associated with progressive reduction in speaking and articulatory rates, and increase in the number and duration of speech pauses [8], [17], [18]. Articulatory rate measures the number of syllables produced in a unit of time and is primarily a measure of speech motor function, influenced by the integrity of the speech musculature (i.e., tongue, jaw, lips, and soft palate). Speaking rate includes both articulatory rate and pausing times, and is a more global measure of speech production. Speaking rate is affected not only by changes in oral musculature but also by an increase in the number and duration of pauses, which may be due to speech motor and/or respiratory deficits in ALS without cognitive impairment.

Reports for non-neurologic populations (e.g., those with lung disease), suggest that speech pausing is also affected by disorders of breathing. Respiratory insufficiency results in shorter than normal breath groups, reduced variability of pauses, and increased pause durations during speech tasks [19]. To our knowledge, pausing patterns during speaking have not been previously described in ALS patients with purely respiratory abnormalities, and respiratory deficit has not been accounted for in studies of speaking and articulatory rates in bulbar ALS [8], [17]. In this study, we examine measures of speaking and pausing with respect to the severity of the respiratory deficit without concomitant bulbar signs for the first time. We also consider the effect of co-existing bulbar and respiratory deficits on speaking and pausing measures.

### Speaking and pausing in FTD

Oral picture description has been used as part of the standard cognitive-linguistic assessment in FTD. A number of studies have documented “fluency” problems in PPA variants during this task [20–23]. Fluency has been inferred by using speaking rate as a proxy, and there is reduced speaking rate in PPA, including FTD-PNFA, but not in FTD-BV [23]. Although pauses have not been measured in most studies, the slow rate has often been attributed to inappropriate pausing resulting from sentence formulation difficulties, word finding difficulties, or distractions from the task. When Wilson et al. [23] considered maximum speaking rate, defined as words per minute for the most rapid sequences of connected words—a measure that would be similar to articulatory rate—patients with FTD-PNFA showed impairment, suggesting a motor speech deficit that is consistent with a diagnosis of AOS. AOS is one of two core diagnostic criteria for FTD-PNFA—either AOS or agrammatism must be present [14]—and would lead to slowing of articulatory rate due to motor abnormalities [24]. This measure has not been evaluated in a passage-reading task in this patient group.

To our knowledge, only one study contrasted pause measurements in patients diagnosed with one of the PPA subtypes—FTD-PNFA or the logopenic variant of PPA (lvPPA)–and healthy controls, performing a reading task [25]. The authors reported increased median pause duration and pause duration variability in FTD-PNFA as compared to healthy controls and those diagnosed with the lvPPA subtype. The differences between pause measures obtained for the two patient groups were not sufficient to aid in diagnostic classification between the two subtypes, however. The authors suggested further evaluation of pause measures obtainable from a reading task, since reading (when preserved) is arguably a cognitively simpler task than self-generated discourse [26] and is also much simpler to analyze using automated methods.

### Speech and pause measures as diagnostic markers

Speaking and pausing measures are typically obtained during speech/ language/ cognitive assessments. Speaking measures (e.g., speaking rate and articulatory rate) have been used in ALS for tracking the progression of bulbar signs [19, 20]. Pausing measures have been suggested as possible diagnostic markers of cognitive changes in dementia, including subtypes of PPA [25], [27]. It is important, however, to examine the effect of primary cognitive-linguistic versus primary motor (bulbar versus respiratory) deficits on these measures side by side, particularly since various motor and cognitive impairments that affect speech and pausing may co-exist in the same patient. This study aims to determine if speaking and pausing while reading aloud differs in patients with ALS who vary with respect to the presence and severity of speech motor and/or respiratory motor deficits, and those with FTD-BV or FTD-PNFA, as compared to healthy controls. Based on the existing literature, we hypothesize the following:

Patients will demonstrate differences from the control participants on measures of speaking and pausing, but will exhibit different impairment profiles. Specifically,

1. Patients with a motor speech deficit due to either bulbar ALS or FTD-PNFA will demonstrate a deficit in speech motor function as measured by speech-based measures. Articulatory rate, which reflects articulatory movement abnormalities, will be particularly sensitive to the identification of speech motor abnormalities in patients with signs and symptoms of bulbar ALS and those diagnosed with FTD-NFPA.
2. Patients with a primary respiratory deficit will be distinguished from those with primary speech motor signs, as they will show normal articulatory rates but shorter than normal speech phrases and longer than normal pauses.
3. Patients with FTD-BV will show normal articulatory rates, since oral motor difficulties are not a feature of this syndrome [13], yet pauses will be longer due to the cognitive-behavioural deficit.

## Materials and Methods

### Participants

This research project and the informed consent forms were approved by the Sunnybrook Health Sciences Centre Research Ethics Board, Toronto, Canada (REB# 207–2007 & 087–2010). Participant consent was recorded on paper-based informed consent forms. All participants provided written informed consent prior to inclusion in the study. The University of Toronto Ethics Review Office approved the storage and analysis of de-identified data (REB# 21132).

A total of 136 participants were included: 33 were controls, 85 were diagnosed with ALS, and 18 with FTD (FTD-BV or FTD-PNFA). Demographic and disease characteristics for all groups are given in Table 1. Participants from all groups reported negative history of communication disorders and speech-language therapy and had completed at least a high school education. Participants with ALS were diagnosed by a neurologist (LHZ) with possible, probable, or definite ALS, as defined by the El Escorial Criteria from the World Federation of Neurology [28]. Sixty-one presented with spinal (limb) and 17 with bulbar onset; the remaining 7 patients had mixed (limb+bulbar) onset. None of the patients reported respiratory-onset ALS. Only those who passed the Montreal Cognitive Assessment (MOCA) [29] and did not have clinically observable changes in cognition were recruited. As part of their clinical assessment, ALS patients completed the ALS Functional Rating Scale—Revised (ALSFRS-R) [30], as well as the pulmonary function test, which supplied the % Forced Vital Capacity (%FVC) measure. Eighteen individuals were diagnosed with FTD (9 with FTD-BV and 9 with FTD-PNFA) by an experienced behavioural neurologist (SEB, TWC, or DTW); diagnoses were based on current criteria (FTD-BV [13]; FTD-PNFA [14]).

**Table 1:** Participants’ demographic and disease characteristics. Values are means plus/minus standard deviations.

	Controls (N = 33)	ALS (N = 85)	FTD-BV (N = 9)	FTD-PNFA (N = 9)
Age (years)	59.21±12.57	59.4±10.01	68.44±8.41	66.44±7.37
Sex (M:F)	13:20	53:32	5:4	5:4
Disease duration (months)	NA	40.37±27.39	62.89±23.59	81.00±52.67
MOCA /30	27.21±2.51	26.44±2.55	26.38±2.45	14.56±6.29
ALSFRS-R/ 48	NA	33.53±6.63	NA	NA

FTD-BV = behavioural variant of FTD; FTD-PNFA = FTD with Progressive nonfluent aphasia.

The controls and participants with ALS were part of a completed larger longitudinal study investigating markers of bulbar disease onset and progression in cognitively normal patients with ALS. This larger study included participants with any type of ALS (spinal and/or bulbar onset; total number of subjects = 145; sessions = 797). ALS participants, and their sessions for which a quality audio recording of the relevant speech sample was available, were identified, and then the latest recording was selected for the analysis. This selection was made to ensure that a reasonable number of ALS patients at an advanced disease stage were included. All available control data were used without exclusions. Participants with FTD-PNFA were part of an on-going longitudinal study investigating language impairments in PPA, and the reading task was temporarily added to the test battery in order to collect data for the present study. Participants with FTD-BV were recruited only to provide a reading sample for the present study.

### Speech sample

All participants read a 60-word paragraph which was at a 5^th^ grade reading level, and which was designed with the purpose of aiding automatic pause boundary detection (the Bamboo passage; see Appendix 1). The passage contains voiced consonants (stops like ‘b’,‘d’) at word and phrase boundaries in order to eliminate misidentification of pauses that can occur during voiceless stops. The participants reviewed the passage for approximately 2–3 minutes prior to reading aloud. Patients with FTD-BV were noted to have multiple (false) starts, and, when lost, were prompted to start again. Patients in other subgroups—particularly ALS and controls—read fluently and without difficulty. Participants were instructed to read the passage aloud at their habitual speaking rate and loudness during recording. High quality digital acoustic recordings were obtained (44 kHz, 16 bit resolution). Recordings were obtained with a high quality professional microphone positioned at a constant distance of approximately 5 cm from the mouth.

### Signal pre-processing

All pre-processing of audio waveforms was done using Adobe Audition^®^ (version 2.0). Each audio recording was reviewed for reading errors, which included misread words (“rapidly” as “rabidly”), word or phrase repetitions (e.g., “We will, oops, we will), extraneous words or phrases (e.g., “I wonder what a bamboo wall is?”), filled pauses (e.g., “um”, “er”), and non-speech vocalizations such as coughing, laughing or sighing. Misread words were included in the analyses without any editing. Word repetitions, phrase or word insertion, and non-speech vocalizations, as well as the pause that immediately followed any of these events, were deleted from the waveforms. Filled pauses were preserved but the waveform amplitude during these events was manually attenuated so filled pauses could be measured as a pause event. In recordings of healthy controls and individuals with ALS, all of these events were rare (<0.5% of total events); so no manual editing was performed for these groups. In the group of patients with FTD, these events were more common. In total, 8% of the text was produced with some type of an error. Combined, the FTD patients made 86 errors on 1080 words (60 words in the passage x 18 patients). Word repetitions and phrase insertions were the most common errors (occurring on 68/1080 words). There were also a few word omissions during readings; we decided not to exclude readings with omissions from the analysis due to their small number (18/1080 words) and random occurrence. To establish reliability for our analysis procedure, 5/18 (27%) recordings obtained from participants with FTD were hand-edited and re-measured by a second judge. The agreements between speech and pause measures obtained from the two analysts were all >95%.

**Fig 1:**
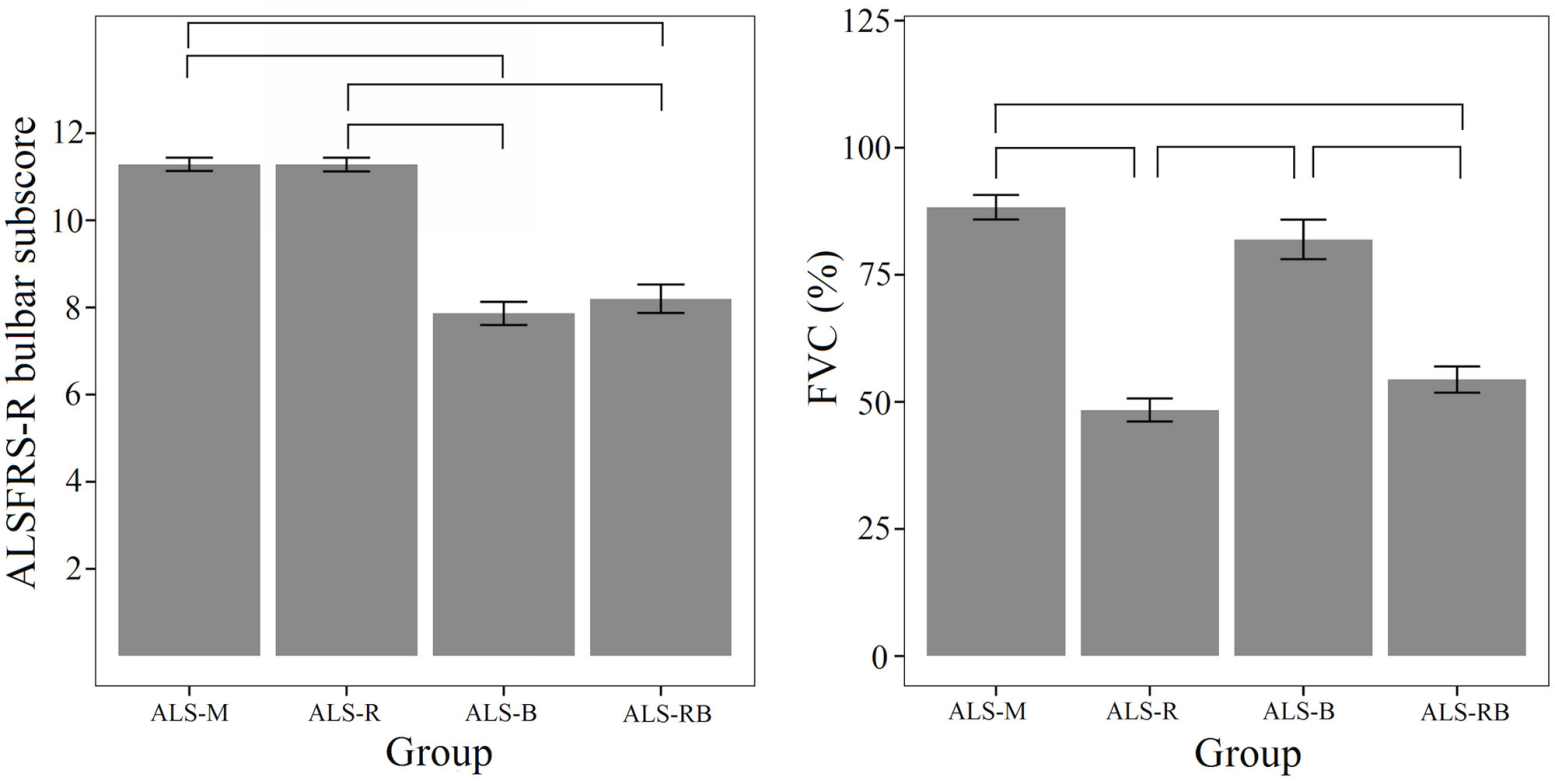
The acoustic waveform recorded from a control participant with pauses—the silent intervals of 300 msec or longer—automatically identified by the SPA software.

After all “non-reading” related events were hand-edited, the audio waveforms were down-sampled to 16 kHz. This audio file was run through Speech Pause Analysis (SPA) software, a semi-automated MATLAB speech pause segmentation procedure [17]. The minimum speech threshold value was set at 25 msec and the minimum pause threshold was chosen to be 300 msec. A previous study on SPA threshold values found that a minimum pause threshold of 300 msec maximized sensitivity and specificity of pause detection by the SPA protocol [30]. As a result, the speech signal boundaries associated with each pause, with below the signal amplitude threshold, were identified on the waveform (see Fig 1). Another listening test was conducted for each recording at this point to verify the accuracy of the pause locations identified by the software.

### SPA measurements

The returned SPA output comprised the time stamps associated with each speech and pause event and their summary statistics. The primary variables of interest included in the analyses were:

A global speech measure:

(1) Speaking rate, in words per minute (WPM)–a measure of overall rate of speaking, which includes articulatory rate and pause time.

Speech-based measures:

(2) Articulatory rate, in syllables per minute (SPM)–a measure of the rate of speaking with pause time automatically removed by SPA. This measure detects articulatory movement slowing which is associated with changes in oral motor function due to disease.
(3) Mean phrase duration, in seconds—represents the average duration of a phrase. Phrases are defined as sections of continuous speech between pauses.
(4) Coefficient of variation of phrase durations (CV phrase)—is a normalized measure of variability of phrase durations.

Pause-based measures:

(5) Pause time, in seconds, is a measure of the total time spent pausing during the entire reading of the passage.
(6) Number of pauses (# of pauses) is the count of times the reader paused while reading the passage.
(7) % Pause is the percentage of total reading time spent pausing.
(8) Mean pause duration in seconds is the average duration of all pauses.
(9) Coefficient of variation of pause duration (CV pause) is a normalized measure of variability in the duration of the pauses (i.e., standard deviation (SD) of all pause durations/mean of all pause durations).

SPA speech-based measures, which evaluated the integrity of speech articulation, including articulatory rate, mean phrase duration and CV phrase, represent the integrity of speech articulation and, therefore, were expected to be sensitive to bulbar motor impairment. The pausing measures, in contrast, were expected to reflect respiratory and cognitive dysfunction, as well as bulbar impairment. Speaking rate could be affected by any or all of these deficits.

### Statistical analysis

Statistical analyses were performed using IBM SPSS Statistics (v.20). Differences in ALSFRS-R and %FVC scores between subgroups of patients with ALS were evaluated using the Mann-Whitney U test and the independent samples t-test, respectively. In order to examine the effect of the underlying deficit on speech and pause measures, group differences were evaluated using an ANOVA, followed by pairwise comparisons, including age and sex as covariates. Pairwise comparisons were performed using Tukey’s HSD test. For the speech and pause measures identified by Levene’s Test of Equality of Variances as having possibly unequal group variances, a non-parametric Welch-Satterthwaite test was used to assess the main effect of group; these measures included mean pause duration and CV phrase. Post hoc multiple comparisons were carried out using the Games-Howell approach [31]. Univariate and multivariate regression analyses were used to evaluate the effect of bulbar and respiratory deficits on SPA measures in the group of patients with ALS. Each regression model was considered to be significant at *p*<0.05.

A canonical linear discriminant analysis (LDA, performed using SPSS) was used to predict whether an individual belonged to the “speech-motor” deficit group, which included ALS-B, ALS-RB and FTD-PNFA, or to the “no-speech-motor” deficit group, which included ALS-R, ALS-M and FTD-BV. The predictor variables were identified from the list of all SPA measures using a stepwise Wilk’s lambda approach. The predictors were selected for entry or removal with a criterion of *F* = 3.84 for entry and *F* = 2.17 for removal. The discriminant analysis used a leave-one-out cross-validation approach to determine the success of the classification model. This cross-validation method categorized each individual observation in relation to the remaining set, repeating the test as many times as there were observations. Classification accuracy, defined as a proportion of cases misclassified, was reported.

## Results

Three outliers were eliminated prior to statistical analysis. One individual in the control group was eliminated because the data were more than 3SDs below those in the same group. Data for two patients with ALS were also removed, as they were more than 3SDs above those obtained for normal controls. As a result, group sizes changed to 32 and 83 for the control and ALS groups, respectively.

### Subgrouping of patients with ALS

In order to examine the effect of the underlying deficit on speech and pausing measures, we analyzed subgroups within the ALS group, which were identified on the basis of the presence and severity of bulbar, and respiratory signs. The patients fell into four subgroups: ALS-B (primarily bulbar), ALS-R (primarily respiratory), ALS-RB (mixed respiratory and bulbar), and ALS-M (mild or absent clinical bulbar or respiratory signs) based on the %FVC, which indicates respiratory status, and the bulbar subscore of the ALSFRS-R, which indicates the severity of bulbar involvement. The presence and severity of spinal (limb) involvement were not considered, as they are not expected to affect speech and pausing measures. Individuals with a score below 10 on the bulbar subscore of the ALSFRS-R but normal or near normal %FVC (>70%) comprised the bulbar subgroup (ALS-B). Individuals with %FVC below 70% and a bulbar score of 10 or above were included in the respiratory subgroup (ALS-R). Those with both %FVC < 70% and bulbar score <10, comprised the respiratory-bulbar subgroup (ALS-RB). Patients with %FVC >70% and bulbar score >10 comprised the mild subgroup (ALS-M). ALS-M patients showed spinal-limb motor deficits of different severity as indicated by the total score on ALSFRS-R, however.

**Fig 2:**
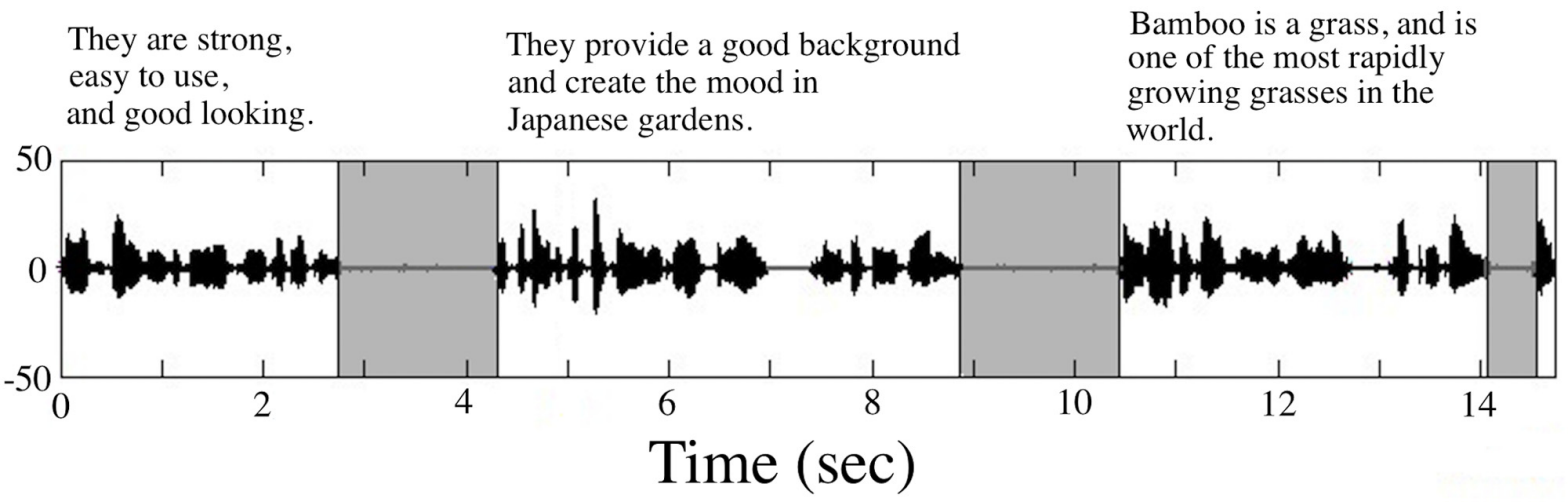
Bar plots showing group differences on the two measures that were used to differentiate patients with ALS into subgroups—ALSFRS-R bulbar subscore and %FVC. Error bars display 1 standard error of measurement (SEM).

Demographic and overall disease characteristics for each ALS subgroup are presented in Table 2. Variables used for patient subgrouping and associated statistics are illustrated in Fig 2. Statistical analyses showed that the bulbar subscore of the ALSFRS-R clearly separated the ALS-M and ALS-R subgroups from the ALS-B (*U* = -5.887, *p*<0.001) and ALS-RB (*U* = -4.762, *p*<0.001) subgroups; %FVC distinguished the ALS-R and ALS-RB from the ALS-M (*t* = -7.780, *p*<0.001) and ALS-B (*t* = -9.023, *p*<0.001) subgroups, as revealed by the Mann-Whitney U and t-tests.

**Table 2:** Demographic and overall disease characteristics for the ALS subgroups. Values are means plus/minus standard deviations.

ALS (N = 85)				
	*ALS-M (N = 28)*	*ALS-R (N = 25)*	*ALS-B (N = 22)*	*ALS-RB (N = 10)*
Age (years)	59.18±10.31	61.44±10.26	57.09±9.71	60.00±10.10
Sex (M:F)	18:10	17:8	10:12	8:2
ALS duration (months)	38.74±21.87	42.46±22.48	31.64±21.15	59.00±50.28
ALSFRS-R, Total /48	37.00±6.08	31.56±6.04	33.32±5.36	29.20±8.08

ALS-M = mild; ALS-R = respiratory; ALS-B = bulbar; ALS-RB = respiratory-bulbar.

### Speech and pause measures by group relative to normal controls

Descriptive statistics for each group and measure are given in Table 3. The omnibus test, indicating a main effect of group, was statistically significant (*p*<0.05) for each measure. The effects of sex and age were not significant in any of the models. The overall measure of speaking rate during passage reading showed that all patient groups, except ALS-M, read significantly more slowly than controls.

**Table 3:** Means and standard deviations on each of the speech and pause measures for each group.

	ALS				FTD		Controls (N = 32)
	*ALS-M (*N *= 27)*	*ALS-R (*N *= 25)*	*ALS-B (*N *= 21)*	*ALS-RB (*N *= 10)*	*FTD-BV (*N *= 9)*	*FTD-PNFA (*N = *9)*	
Speaking rate (WPM)	156.63 ±27.96 ^c^,^d^,^f^	150.02 ±24.87** ^c^	104.43 ±26.27*** ^a^,^b^,^e^	123.45 ±38.73*** ^a^	140.45 ±48.88* ^c^	118.57 ±32.15*** ^a^	176.83 ±20.93
Articulatory rate (SPM)	268.53 ±37.64^c^,^f^	267.93 ±37.80^c^,^f^	176.30 ±38.90***^a^,^b^,^d^,^e^	234.17 ±67.32***^c^	256.32 ±47.32^c^	219.04 ±35.34***^a^,^b^	285.79 ±29.85
Mean phrase duration (sec)	2.85 ±0.85	2.31 ±0.93 **	2.91 ±0.63	2.05 ±0.50**	2.87 ±1.95	2.08 ±0.69**	3.19 ±0.55
CV phrase†	0.67 ±0.17***	0.59 ±0.10***	0.57 ±0.19***	0.57 ±0.16*	0.53 ±0.23	0.61 ±0.17**	0.39 ±0.11
Pause time (sec)	8.19 ±4.21	9.62 ±7.45	12.36 ±3.56*	15.69 ±8.25**	17.10 ±21.39***	15.82 ±12.76**	5.19 ±1.50
# of Pauses	11.11 ±4.16	13.60 ±8.44	16.90 ±4.88**	19.20 ±7.44**	19.56 ±21.62**	20.11 ±10.18**^a^	8.44 ±1.98
% Pause time	20.06 ±7.11	23.17 ±6.51**	19.94 ±5.61	27.89 ±6.47***	25.91 ±19.06 **	26.18 ±12.93**	15.07 ±3.15
Mean pause duration (sec)†	0.72 ±0.17	0.72 ±0.17	0.71 ±0.16	0.79 ±0.20	0.76 ±0.20	0.73 ±0.32	0.62 ±0.12
CV pause	0.38 ±0.18	0.34 ±0.20	0.43 ±0.13**	0.45 ±0.18*	0.50 ±0.28**	0.44 ±0.18	0.26 ±0.08

ALS: M = Mild, R = Respiratory, B = Bulbar, RB = Respiratory-Bulbar; FTD: FTD-BV = Behavioral variant FTD, FTD-PNFA = Non-fluent primary progressive aphasia, CV = coefficient of variation, WPM = words per minute, SPM = syllables per minute. Asterisks denote significantly impaired relative to normal controls at

*p<0.05;

**p<0.01;

***p<0.001. Superscript letters denote significantly impaired relative to the

^a^ mild,

^b^ respiratory,

^c^ bulbar,

^d^ respiratory-bulbar,

^e^ FTD-BV, and

^f^ FTD-PNFA at *p*<0.05.

^†^Tested with non-parametric statistics.

Among speech-based measures, articulatory rate was impaired in ALS-B, ALS-RB, and FTD-PNFA relative to controls, while the ALS-M, ALS-R, and FTD-BV groups showed normal performance. Shorter mean phrase durations were observed in patients with ALS-R and ALS-RB, as well as FTD-PNFA, as compared to those in the control group, while the ALS-M, ALS-B, and the FTD-BV groups showed normal mean phrase durations. All patient groups except FTD-BV showed statistically larger-than-normal variability in phrase durations (CV phrase), and this was the only measure that distinguished the ALS-M group from healthy controls.

When pausing behaviors were considered, the measures of total pause time and the number of pauses were significantly impaired in patients in the ALS-B, ALS-RB, FTD-BV, and FTD-PNFA groups, but these two measures did not distinguish ALS-M or ALS-R from normal controls. % Pause time was elevated with respect to controls in all but the ALS-M and ALS-B groups. Mean pause duration was equivalent across all groups. Greater CV pause was observed for ALS-B, ALS-RB, and FTD-BV patient groups, as compared to normal controls.

### Speech and pause measures—Comparisons between patient groups

The only measures that distinguished the disordered groups from each other were speaking rate and articulatory rate (see Table 3). Speaking rate separated ALS-B from ALS-M (d = -1.92), ALS-R (d = -1.78), and FTD-BV (d = -0.92), with bulbar patients showing slower speaking rates than those with a purely respiratory deficit and those with FTD-BV. Articulatory rate revealed significant differences between ALS-B and the ALS-M, ALS-R, ALS-RB, and FTD-BV groups, with the ALS-B group showing a significant articulatory rate reduction relative to all the other groups (with effect sizes for the significant differences of -2.41–2.39, -1.05, and -1.85, respectively). Although the articulatory rate in the FTD-PNFA group was statistically equivalent to that of the ALS-B group, there was no significant difference between the FTD-BV and FTD-PNFA groups. The remaining speech and pause measures did not distinguish the disordered groups from each other.

### Speech and pause measures and bulbar versus respiratory deficits in ALS

**Fig 3:**
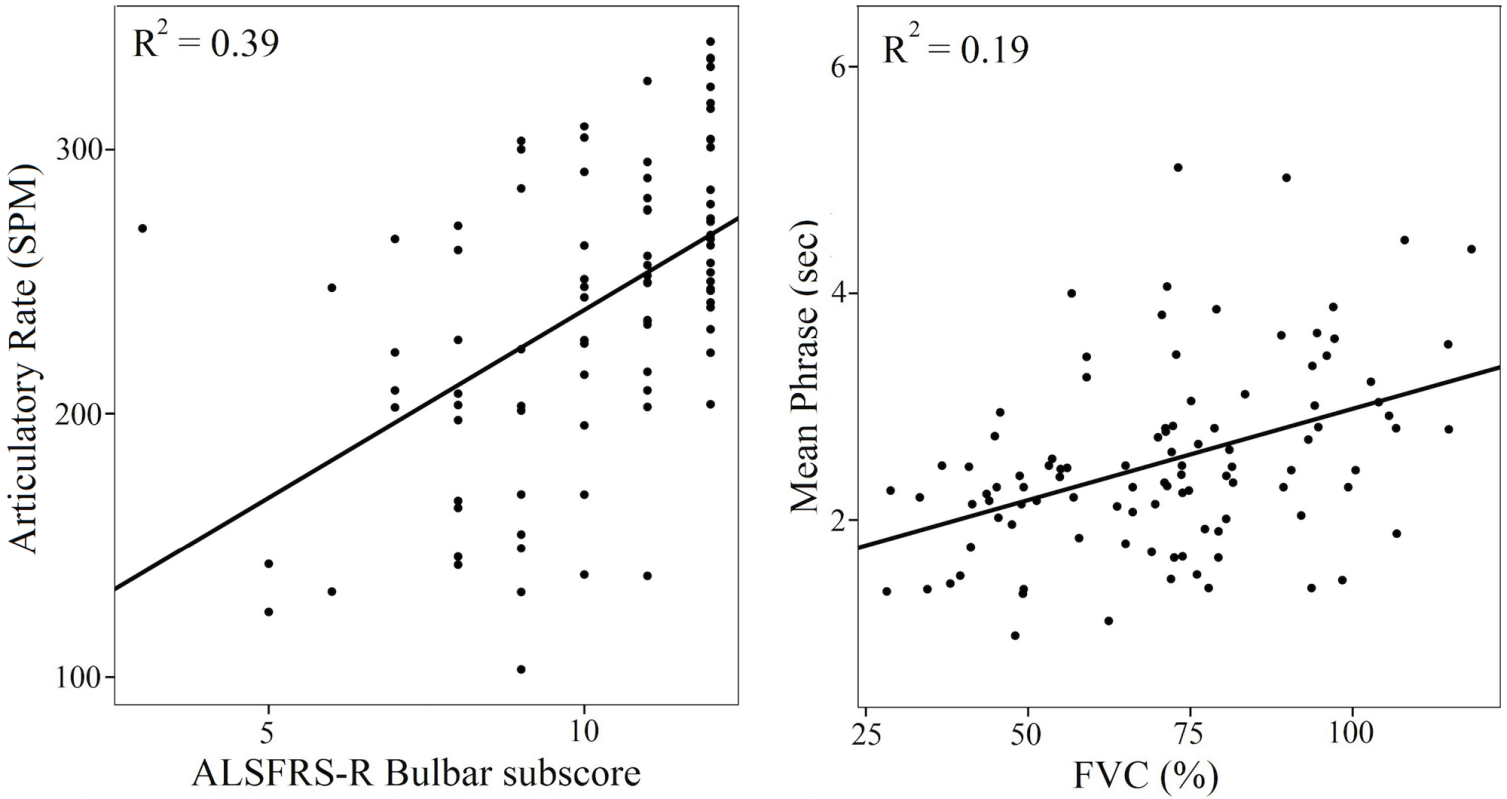
Correlations between (a, left) ALSFRS-R bulbar subscore (/12) and articulatory rate (SPM); and (b, right) FVC (%) and Mean phrase durations (sec) with the coefficients of determination in patients with ALS.

Regression analyses were used to assess the contribution of the bulbar and/or respiratory motor impairment to speech and pause measures. Data from all ALS patients were used in the regression (N = 83). The results are shown in Table 4 and selected plots in Fig 3. The ALSFRS-R bulbar subscores were significantly associated with speaking and articulatory rates, pause time, # of pauses, and CV pause. %FVC scores were significantly associated with articulatory rate, mean phrase duration, and % Pause time. Multiple regression analyses examined the contribution of both clinical measures—bulbar subscore and %FVC—to speech and pause measures. They revealed that, when controlling for %FVC, the bulbar subscore significantly contributed to measures of speaking and articulatory rates, pause time, # of pauses, and CV pause. Controlling for the bulbar subscore, %FVC contributed significantly to % pause time, mean phrase duration and CV phrase.

**Table 4:** Coefficients of determination (R^2^) from regression analyses assessing contribution of bulbar and/or respiratory impairments to performance on each speech and pause measure; N = 83.

Variable	~ALSFRS-R Bulbar subscore	~% FVC	~Bulbar subscore + %FVC
Speaking rate	0.36***	0.01	0.36***
Articulatory rate	0.39***	0.05*	0.41***
Mean phrase duration (sec)	0.01	0.19***	0.20***
CV phrase	0.03	0.04	0.09*
Pause time (sec)	0.17***	0.01	0.20
# of Pauses	0.16***	0.02	0.20***
% Pause time	0.01	0.13***	0.15***
Mean pause duration (sec)	0.02	0.01	0.03
CV pause	0.08*	0.02	0.09*

^~^ Predictor variable in the model; asterisks denote significance at

*p<0.05;

***p<0.001.

### SPA measures and speech-motor versus no-speech-motor deficits in neurodegenerative diseases

When all groups were re-coded into “speech-motor” deficit (ALS-B, ALS-RB, FTD-PNFA) and “no-speech-motor” deficit (ALS-R, ALS-M, FTD-BV) categories, LDA showed that articulatory rate was the only variable that was able to discriminate between these two patient categories. The structure matrix revealed a correlation of 0.99 between articulatory rate and the discriminant function. The measure of articulatory rate accounted for 34.8% of the variance in the model. The leave-one-out cross-validated classification showed that 78.2% of cases were classified correctly. The “no-speech-motor” group was classified with greater accuracy (90.2%) than the “speech-motor” group (60.0%). When the ALS-RB group was removed from the “speech-motor” group to eliminate the contribution of the respiratory deficit, the overall classification accuracy of the model increased to 85.7%. The “no-speech-motor” group was classified with 93.4% accuracy, while the “speech motor” group was classified with 70.0% accuracy.

## Discussion

### Summary

Speech and pausing behaviors during reading aloud were examined in this study for patients with primary motor (i.e., speech and respiratory) deficits due to ALS, vs. patients with primary cognitive-linguistic deficits due to FTD-PNFA or FTD-BV, and in comparison with normal controls. Clear differences emerged between patient groups and controls and between the different patient groups, indicating differential effects of the underlying deficit on speech and pausing during reading. Speech-based measures, particularly the articulatory rate, were able to distinguish patients with a speech-motor deficit (bulbar ALS or FTD-PNFA) from those with a respiratory deficit in ALS or FTD-BV. Distinguishing among the disordered groups proved challenging based on the pausing measures alone as pauses were affected equally by motor or cognitive-linguistic etiologies.

### Do speech and pause measures have diagnostic value?

Speaking rate is commonly used as a global measure in the assessment of bulbar ALS and various forms of FTD [16], [18], [23], [32]. In our study, speaking rate was impaired in all but the mild ALS patients, confirming its high sensitivity to these disease states. As a relatively complex phenomenon, subsuming both articulatory rate, which reflects the articulatory movement speed, and pausing, which might be indicative of respiratory, language (e.g., word finding, phonological encoding) or cognitive (e.g., initiation, formulation) abnormalities [33], [34], speaking rate can be impaired in a variety of brain diseases including Alzheimer’s disease (AD), Parkinson’s disease (PD), depression, and schizophrenia [35–38]. Thus, when used by itself, speaking rate provides limited insight into the nature of the underlying impairment and must be examined in conjunction with other measures.

As hypothesized, articulatory rate emerged as a predictive measure of motor speech deficit as it indicated motor speech abnormalities in the ALS-B, ALS-RB and FTD-PNFA but not in ALS-M, ALS-R or FTD-BV groups. The finding for FTD-PNFA was consistent with reports of the motor speech disorder of AOS, which is currently a core diagnostic feature of FTD-PNFA [14], [39], [40]. Recent studies, however, also suggested that reduction in articulatory rate might be associated with the presence of cognitive impairment [41], [42]. Specifically, Rodgers, et al. [42] found that information processing speed, but not memory or other executive measures, accounted for about 30% of variance in speaking and articulatory rates in reading and narrative tasks in patients with multiple sclerosis (MS). Although it is an intriguing finding, as evidence of cognitive-motor interactions is emerging in recent literature [43–45], more work needs to be done to fully understand its basis. Patients with ALS, for example, show normal processing speeds in the face of significantly affected articulatory rates [46]. Further work in neurodegenerative diseases of various etiologies with specific motor and cognitive abnormalities will help to determine the nature of cognitive-motor interactions in the control of speech production.

In our study, pause measures were often impaired across all disordered groups, with the exception of ALS-M and, to some extent, ALS-R. The impaired patient groups showed more and longer pauses as well as higher pause duration variability, in agreement with previous studies [17], [25]. Healthy speakers spent on average only 15% of their reading time on pausing, while the impaired groups spent approximately 25% of their reading time on pausing. Many other neurologic disorders affect pausing behaviors during speaking, including traumatic brain injury, PD, AD, and MS [41], [47], [48]. Although suggestions of using pausing as a diagnostic indicator have been voiced in FTD [25], [27], there may not be enough difference between disorders of various origins [25] with respect to their effects on pausing, and multivariate approaches will be necessary to devise a diagnostic assessment with high sensitivity and specificity.

### Which measures distinguish “speech-motor” from “no-speech-motor” deficits?

The presence of articulatory abnormalities was clearly identified by the measure of articulatory rate in the group of patients with bulbar ALS and FTD-PNFA, which supports the assertion that the measure of articulatory rate has diagnostic value in detecting speech motor changes. Our prediction did not hold with respect to other speech-based measures. When individual speech phrases were considered (e.g., mean phrase duration), the differentiation between “speech motor” and “no-speech-motor” deficits was less clear, most likely because, during reading, speakers are generally free to vary the duration of speech phrases. For example, in our sample those with bulbar ALS produced phrase durations that were similar to healthy controls, despite the fact that they also produced almost twice as many speech phrases as those in the control group (the number of speech phrases can be inferred from the measure of # of pauses). Pause measures, as predicted, did not distinguish the “speech motor” from the “no-speech-motor” deficit groups and, instead were affected across most patient groups.

### Do speaking and pausing profiles differ in patients with ALS with primary bulbar motor vs. respiratory symptoms?

As hypothesized, patients with respiratory symptoms due to ALS showed normal articulatory rate but shorter phase durations and larger % pause time. Only speaking and articulatory rates differed significantly between those with primary bulbar versus primary respiratory involvement in ALS, suggesting that articulatory rate—a component of speaking rate—is the primary differentiator. However, in many patients with ALS, the bulbar and respiratory deficits co-occur, as in the ALS-RB group. We attempted to differentiate the bulbar versus the respiratory effects using regression analyses. These analyses revealed that different measures were associated with variation in bulbar subscores of ALSFRS-R versus %FVC, gold-standard clinical measures of functional decline in ALS, and respiratory impairment, respectively. Changes in measures of speaking and articulatory rates, pause time, # of pauses and the CV of pause duration were primarily linked to the presence and severity of bulbar deficit. Respiratory abnormalities explained the greatest variability in % time spent pausing, average duration of a speech phrase, and the coefficient of variation of phrase duration. These data suggest that a simple reading task and the SPA assessment method—for which an online module is under development—may be used by speech language pathologists to monitor changes not only in bulbar, but also in respiratory performance, as part of the clinical management of ALS.

### Which measures differentiate FTD-BV from FTD-PNFA?

As predicted, articulatory rate clearly distinguished patients with FTD-PNFA from healthy controls, but it did not distinguish the FTD-PNFA and FTD-BV groups. In fact, surprisingly, none of the speech and pause measures clearly separated the two FTD groups from each other. On the speech-based measures (i.e., articulatory rate, mean phrase duration, and CV phrase), the impairment showed the following (non-significant) pattern: FTD-PNFA > FTD-BV > controls. Although the FTD-BV group showed normal performance on the motor measures (e.g., see CV phrase) while the FTD-PNFA group was impaired, there were no statistically significant differences between these groups. This might be due to the small sample sizes of the FTD groups and the large between-subject variability. Alternatively, this observation may be due to the inherent association between increased pausing and its effect on slowing of the articulatory rate, as has been reported in healthy controls [8]. In our sample of the control participants, the correlation between articulatory rate and pause time was *r* = -0.50, indicating that, among healthy readers, those who paused more tended to speak with slower articulatory rate. For comparison, these correlations were *r* = -0.58 and *r* = -0.43 for the combined ALS and FTD groups, respectively. Alternatively, the lack of distinction between the FTD-BV and FTD-PNFA groups may be due to the nature of the task—the performance of both groups could be affected by a behavioural and/or language/reading deficit.

### Detection of early changes in ALS

Only one measure–CV phrase, which represented the variability of phrase durations during reading—distinguished the ALS-M group from healthy controls. This finding indicates that the early onset of difficulty in planning and controlling speech breathing is evident even in such a simple task as paragraph reading. This is an interesting finding as it points to the possibility of using this measure for predicting clinical changes in bulbar or respiratory functions at the later stages of disease. Because early detection of bulbar changes is a high priority in ALS-related research, both for diagnostic purposes and for patient subgrouping for clinical trials, this finding warrants further investigation in a longitudinal study.

### Using SPA as a method of speech and pause data analysis

Speech and pause analyses are very labor intensive but clearly useful in describing the performance of individuals with various neurodegenerative conditions. These analyses could indicate changes in performance early in the course of the illness and assist in monitoring disease progression. There are a number of technical/ methodological developments in the areas of pause boundary identification and speech/ breath group segmentation [17], [49], [50]. Regardless of the specifics of the approach, these methods are comparable to live operator performance, but faster and easier to perform.

However, there are challenges involved in using a reading task and automated analysis in patients with primarily cognitive-linguistic deficits and caution is warranted during data collection and analyses. One challenge is that patients with FTD may present with a reading deficit [51–53]. None of the patients in this study exhibited a severe problem in reading as they were able to complete the task, and the number of reading errors was small in our data (note that certain errors such as word/phrase repetitions and inclusion of fillers were easily edited out during pre-processing). If a reading task is used for this purpose in the future, then, ideally, the reading abilities of the patients should be formally assessed using standardized reading tests. Alternatively, SPA can be performed on a conversational speech task. A careful operator-driven data examination should be performed in this case as the SPA algorithm may be challenged by certain sounds at pause boundaries [17]. Another challenge is that PPA syndromes are associated with language deficits (e.g., phonological, morphological), resulting in various errors in speech production (e.g., sound omissions, insertions, substitutions), any of which could have an effect on timing measures. Future studies will have to address the development of a simplified reading passage, which would avoid irregular or rare words, and develop adaptations for sound or word omissions. Alternatively, linguistic error analyses could be built into the software, providing a more detailed assessment of the deficit associated with FTD subtypes.

## Limitations

Limitations of this study need to be considered when interpreting its results. First, our patient groups were unbalanced in that the FTD groups consisted of a relatively small number of participants. Additionally, a more detailed assessment of associated cognitive and language/reading abilities should be performed in future studies across all of the participants. Furthermore, a more detailed analysis of errors in reading should be developed to expand on quantification of speech and pause events in SPA.

## Conclusions

From a clinical perspective, this study demonstrated the usefulness of performing a relatively simple reading test with an algorithmic method of assessing speech versus pause behaviors across the ALS—FTD disease continuum. It is clear that motor speech assessment should be performed in patients with FTD to identify speech motor abnormalities (see [54]). Caution needs to be applied in interpreting these measures, however. Further work is required in the domain of cognitive-motor interactions to fully explain how changes in motor control affect cognitive indicators, and vice versa, and why they often emerge at the same time or co-occur in the same individual.

## Appendix 1

Bamboo walls are getting to be very popular. They are strong, easy to use, and good looking. They provide a good background and create the mood in Japanese gardens. Bamboo is a grass, and is one of the most rapidly growing grasses in the world. Many varieties of bamboo are grown in Asia, although it is also grown in America. Last year we bought a new home and have been working on the flower gardens. In a few more days, we will be done with the bamboo wall in one of our gardens. We have really enjoyed the project.
